# Chemometric approach for discriminating tobacco trademarks by near infrared spectroscopy

**DOI:** 10.1016/j.forsciint.2018.10.016

**Published:** 2019-01

**Authors:** Jone Omar, Boleslaw Slowikowski, Ana Boix

**Affiliations:** European Commission, Directorate-General Joint Research Centre, Directorate F – Health, Consumers and Reference Materials, Retieseweg 111, 2440 Geel, Belgium

**Keywords:** Near infrared spectroscopy, Tobacco, Chemometrics

## Abstract

Cigarettes are consumer products with a broad market-driven orientation. In order to satisfy the different needs of smokers, cigarette trademarks with different aroma, taste and appearance are available on the market. In this study near infrared (NIR) spectroscopy in combination with chemometrics was applied in order to distinguish cigarette trademarks by analysing the tobacco. Calibration models were developed to differentiate three tobacco trademarks and their respective producers. The developed models also allowed the discrimination of the tobacco according to their geographical origin and may serve as a tool for the detection of counterfeit tobacco.

## Highlights

• Near infrared spectroscopy: a non-destructive method for discrimination of tobacco trademarks.
• Chemometrics enhance the potential of spectroscopy for the discrimination.
• A potential tool for tracing tobacco trafficking routes and detecting counterfeit tobacco.

## 1 Introduction

Tobacco is a complex product because it is represented by several species belonging to the *Nicotiana* genus; however, mainly five species are used in commercialised tobacco. The chemical composition of those species differs and commercial tobacco products are blends of the leaves of different species cured in different ways [1]. Apart from the variability in tobacco species and processing techniques, 600–1400 additives are added to tobacco, making it a complex matrix to be analysed [2]. These additives together with the specific blend of tobacco types account for the uniqueness of each trademark of cigarettes determining their aroma and taste. Trademark discrimination by physical-chemical methods is possible but is mostly done by human sensory responses, which are time consuming and subjective to a certain degree [3]. Properties of cigarettes can also vary from batch to batch of the same trademark because of the compositional variation of the tobacco leaves from different harvests, growing regions, etc. [4].

Tobacco is a globally available consumer product, which is regulated and taxed in all countries around the world. This creates the opportunity for illegal activities to evade taxation and customs duties [5]. According to estimates of the WHO Framework Convention on Tobacco Control the trade in illicit cigarettes causes losses of 30 billion US$ to the revenues of national administrations [6]. Moreover, counterfeit products can have potentially increased health hazards due to uncontrolled production processes or treatments. To facilitate the smooth functioning of the internal market for tobacco and related products, the European Commission issued the Tobacco Products Directive in 2014 laying down rules for the manufacture, presentation and sale of tobacco products [7]. This Directive introduces, among others, changes in the design of the tobacco packs (i.e. larger health warning messages, eliminates small packages) and introduces tracking and tracing systems (i.e. unique identifier, holograms) to combat illicit trade of tobacco products.

Forensic science can support the fight against illicit cigarettes by creating objective evidence regarding the provenance of seized cigarettes to inform investigators whether the cigarettes are authentic trademarks smuggled from a low-tax to a high-tax jurisdiction, are counterfeit, or “cheap-whites”.

The most commonly used analytical techniques applied for distinction of tobacco are X-ray fluorescence spectroscopy, inductively coupled plasma-optical emission or mass spectrometry and gas chromatography, which require a thorough sample preparation [2], [8]. Vibrational spectroscopy together with chemometrics is a fast, simple and non-destructive tool that has been widely used for characterising medicines, drugs or tablets for many years [9], [10], [11]. It has also been demonstrated to be powerful for the analysis of tobacco [1], [4], [12], [13]. Another advantage of spectroscopy is that the measurements can be performed using portable devices allowing in situ quality control [14].

This paper explores whether near infrared (NIR) spectroscopy combined with chemometrics is a useful tool for discriminating different trademarks of cigarettes manufactured by different producers. The second objective was to find out whether the NIR spectra could be used for revealing the geographical regions where the cigarettes were produced, which would allow tracing trafficking routes. The third objective was to use the developed model to characterise counterfeit tobacco.

## 2 Materials and methods

### 2.1 Sample preparation

200 packs of three globally available cigarette trademarks (Marlboro, Camel and Lucky Strike) were selected to perform this study. Camel is produced by Japan Tobacco International (JTI); Lucky Strike by British American Tobacco (BAT) and Marlboro Red by Philip Morris International (PMI). The cigarettes were purchased at licensed tobacco retailers in various countries (Table 1); out of the 200 packs, 146 came from European countries. An overview of the EU and non-EU cigarette samples used in the study is presented in Table 2. For the same tobacco trademark, the pictograms, printing style, wrapping material etc. varied from country to country, which enhanced the need of creating a database of the tobacco itself. In addition to this sample set, 11 tobacco packs of less known trademarks from China (Daqianmen, Double Happines, Hong Fu Rong, Huanghelou, etc.) were included in the study.

**Table 1:** Summary of the 55 countries where tobacco packs were purchased with the corresponding ISO country codes used for creating the models.

Country	N_o_	ISO	Country	N_o_	ISO	Country	N_o_	ISO
Algeria	1	DZ	Hungary	1	HU	Poland	1	PL
Armenia	1	AM	Iceland	3	IS	Portugal	3	PT
Austria	9	AT	India	1	IN	Senegal	1	SN
Bahamas	2	BS	Iraq	2	IQ	Serbia	3	RS
Belgium	8	BE	Ireland	2	IE	Slovakia	3	SK
Bulgaria	1	BG	Israel	2	IL	Slovenia	1	SI
Cambodia	3	KH	Italy	13	IT	South Africa	2	ZA
Canada	2	CA	Jordan	1	JO	Spain	13	ES
China	6	CN	Lao PDR	1	LA	Sweden	2	SE
Costa Rica	1	CR	Latvia	2	LV	Switzerland	4	CH
Croatia	1	HR	Lebanon	3	LB	Tajikistan	1	TJ
Czech R.	9	CZ	Lithuania	6	LT	Turkey	3	TR
Denmark	2	DK	Luxembourg	4	LU	Ukraine	2	UA
Egypt	2	EG	Macedonia R.	1	MK	United Kingdom	7	GB
Estonia	2	EE	Mexico	1	MX	United States	11	US
Finland	4	FI	Morocco	1	MA	Uzbekistan	1	UZ
France	10	FR	Myanmar	1	MM	Vietnam	3	VN
Germany	12	DE	Namibia	3	NA			
Greece	5	GR	Netherlands	6	NL

**Table 2:** Summary of tobacco packs (samples) used for creating calibration models, from three different tobacco producers: JTI, BAT and PMI; and from three different brands: Camel, Lucky Strike and Marlboro Red.

	Japan Tobacco International (JTI)	British American Tobacco (BAT)	Philip Morris International (PMI)
	Camel	Lucky Strikes	Marlboro Red
EU	47	30	69
Non-EU	11	2	41
Total	58	32	110

The analysis of tobacco required the production of a solid pellet. For the preparation of the tobacco pellets, the tobacco from 12 to 14 cigarettes per pack was collected and dried in an oven at 72 °C for one day. The tobacco was pulverised in a planetary mill (Panalytical MINIMILL 2 - P6 (Almelo, Netherlands)) using a zirconium oxide grinding bowl of 250 ml with zirconium oxide grinding balls (6 × 30 mm). Grinding was undertaken typically for 180 s to fully pulverise and homogenise the material. Approximately 7 g of fine powder were obtained which was sufficient to make a pellet without the need of a binder. The grinding bowl and the grinding balls were thoroughly cleaned between samples to avoid cross contamination. The pulverised tobacco was pressed at 20 t into 40 mm pellets in collapsible aluminium cups, by a Herzog Manual Press TP40 (Osnabrück, Germany). If the pellets are kept neat and dry in a desiccator, they can be stable for at least three months without any sign of physical or chemical degradation [15].

### 2.2 Near infrared spectroscopy

In this study an integrating sphere coupled to a Vertex II spectrometer (Bruker, Germany) controlled by the OPUS (version 7.5) software was used to analyse the tobacco pellets. Three replicates were measured for each tobacco pellet; the final spectrum was an average of the three spectra which were acquired under the following conditions: 64 scans, 4 cm^−1^ resolution, 7600–3900 cm^−1^ wavenumber range. However, for practical reasons the wavenumber region between 7600–3900 cm^−1^ was used in order to avoid unnecessary noise in the models. After each of the samples was measured, a background measurement was carried out with a gold standard.

### 2.3 Data treatment and chemometrics

Before chemometric computation was performed with the acquired data using The Unscrambler^®^ X software (CAMO, Norway), Standard Normal Variate (SNV) transformation was applied to the NIR spectra as general pre-processing step for reducing the differences in intensities among the acquired spectra [11].

Principal Component Analysis (PCA) and Partial Least Square-Discriminant Analysis (PLS-DA) were applied in this study for dimensionality reduction and classification. PCA is an unsupervised method which allows the exploration of the data by performing linear combinations of the initial variables reducing its dimensionality. The data are then represented in the new principal components (PCs) in which the differences among the spectra are enhanced forming clusters [11]. The PCA was carried out with all samples present in Table 1 and the corresponding IR spectra in the range of 7600–3900 cm^−1^. PLS-DA is a supervised model, which maximises the variance between the classes and estimates the distance of a sample from the mean of a set of samples [16]. Both chemometric tools are nicely summarised for the application of tobacco ashes by Pérez-Bernal et al. [17].

## 3 Results and discussion

### 3.1 NIR spectra of tobacco pellets, discrimination of tobacco trademarks

**Fig. 1:**
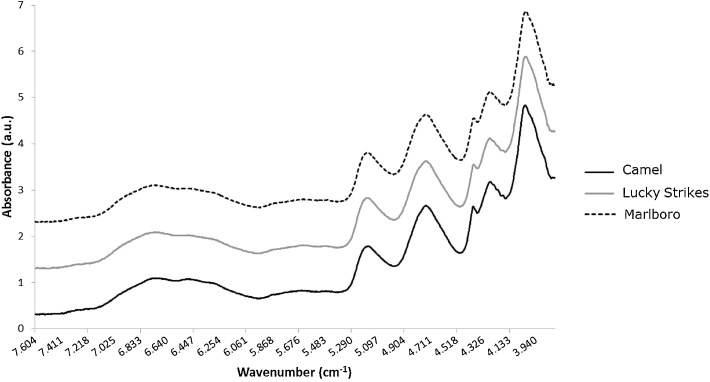
Representative NIR spectra of tobacco pellets in the region of 7600–3900 cm^−1^ from trademarks Camel (JTI), Lucky Strike (BAT) and Marlboro Red (PMI).

A representative spectrum from each of the tobacco trademarks is plotted in Fig. 1. Visually, all the spectra looked very similar and no distinction between trademarks was possible; however, this can be achieved by applying PCA.

**Fig. 2:**
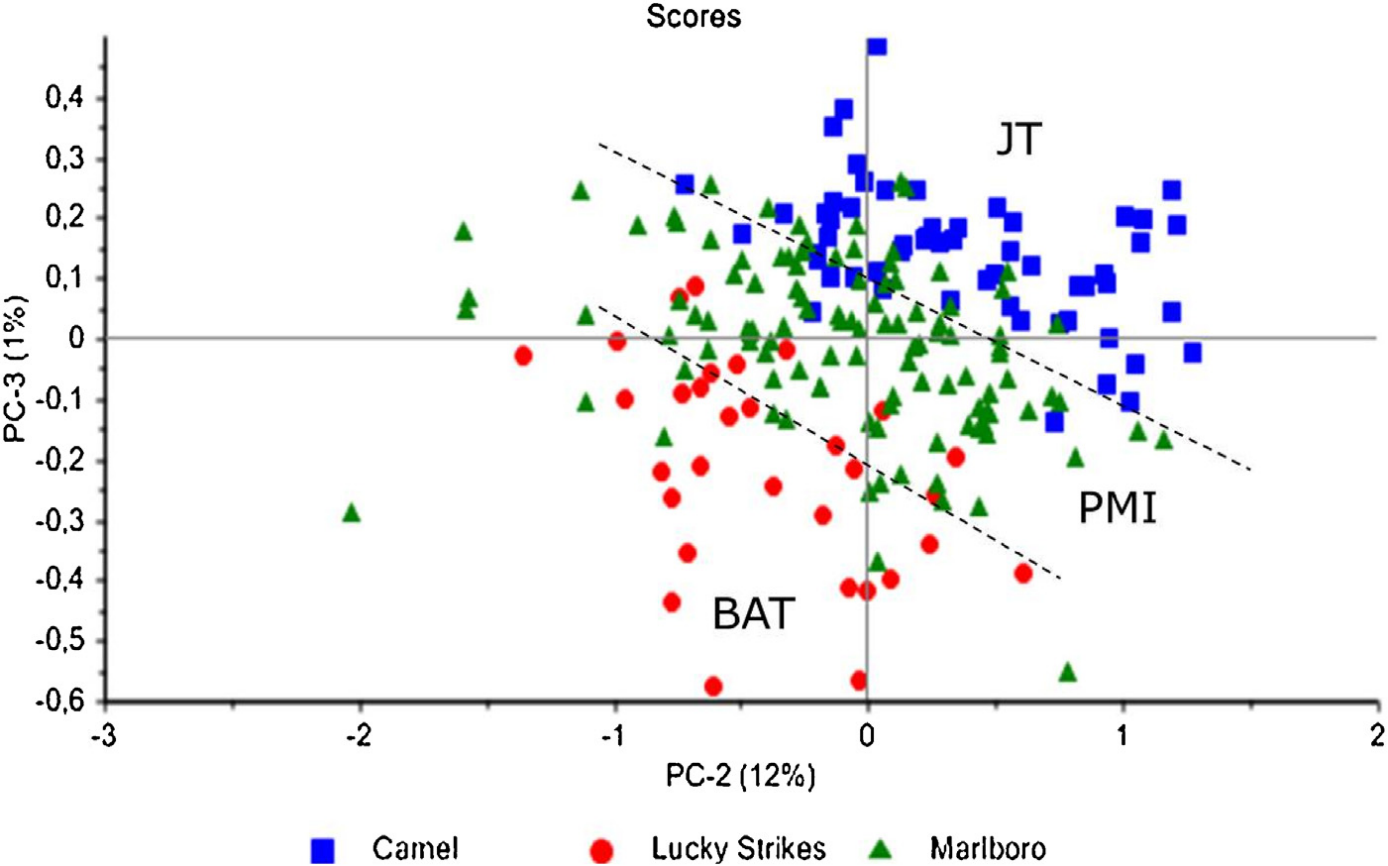
Score plot of PC2 versus PC3 of NIR spectra from three tobacco trademarks: Camel produced by JTI, Lucky Strike produced by BAT and Marlboro Red produced by PMI.

The PCA was carried out with all IR spectra of the samples present in Table 1 after being pre-processed by SNV, the wavenumber region employed in the PCA was 7600–3900 cm^−1^. By using a reduced area of the spectra unnecessary noise is kept out of the model. Each point in Fig. 2 is the average spectra of three tobacco measurements from a cigarette pack with a different geographical origin. Fig. 2 shows the score plot of the PCA using PC2 and PC3, samples from the same trademark are indicated with the same colour, i.e. Camel as blue squares, Lucky Strikes as red circles and Marlboro as green triangles. This PCA, explaining roughly 13% of the total variance shows the clustering of samples the three trademarks obtained from all over the world. PC1 and PC2 (figure not shown) explained even 98% of the total variance but most probably PC1 is projecting the variation in the major components of tobacco, such as: cellulose-lignin, sugars, nicotine, etc. More subtle differences shown by PC2 and PC3 appeared to be responsible for trademark discrimination.

**Fig. 3:**
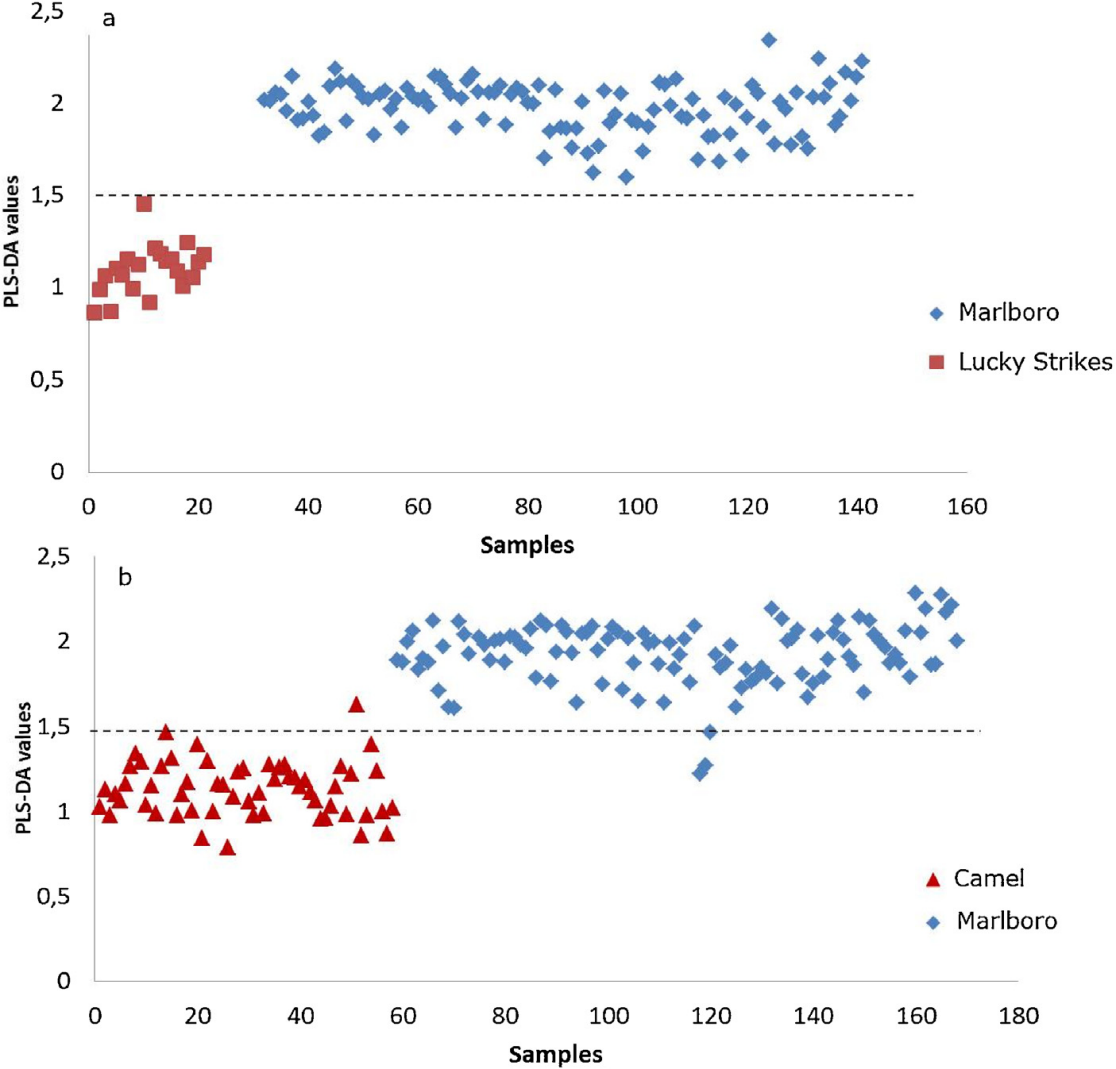
Sample grouping of PLS-DA, (a) tobacco companies BAT and PMI; (b) tobacco companies JT and PMI. The response variables were set to 1 and 2 respectively.

In order to confirm the trend observed by the PCA, PLS-DA was employed to differentiate among trademarks and/or companies, i.e. PMI vs JT and PMI vs BAT. The response variable was set to a value of 1 for Lucky Strikes (BAT) samples; to a value of 2 for Marlboro (PMI) samples in the first PLS-DA Fig. 3a, while Camel (JT) samples were set to a value of 1 and to a value of 2 for Marlboro (PMI) samples, see Fig. 3b. In both cases a perfect separation between both trademarks was achieved as observed in Fig. 3. The models were obtained by employing the first 6 factors, subjected to random cross validation (20 segments). For the case of PMI vs BAT (Marlboro vs Lucky Strikes, Fig. 3a) both companies are clearly distinguished, while in the case of PMI (Marlboro) vs JT (Camel) just five out of 168 samples seem to be misallocated (at a 95 % confidence level), which underlines the robustness of the method.

### 3.2 Discrimination of geographical origin of tobacco from the same producer

**Fig. 4:**
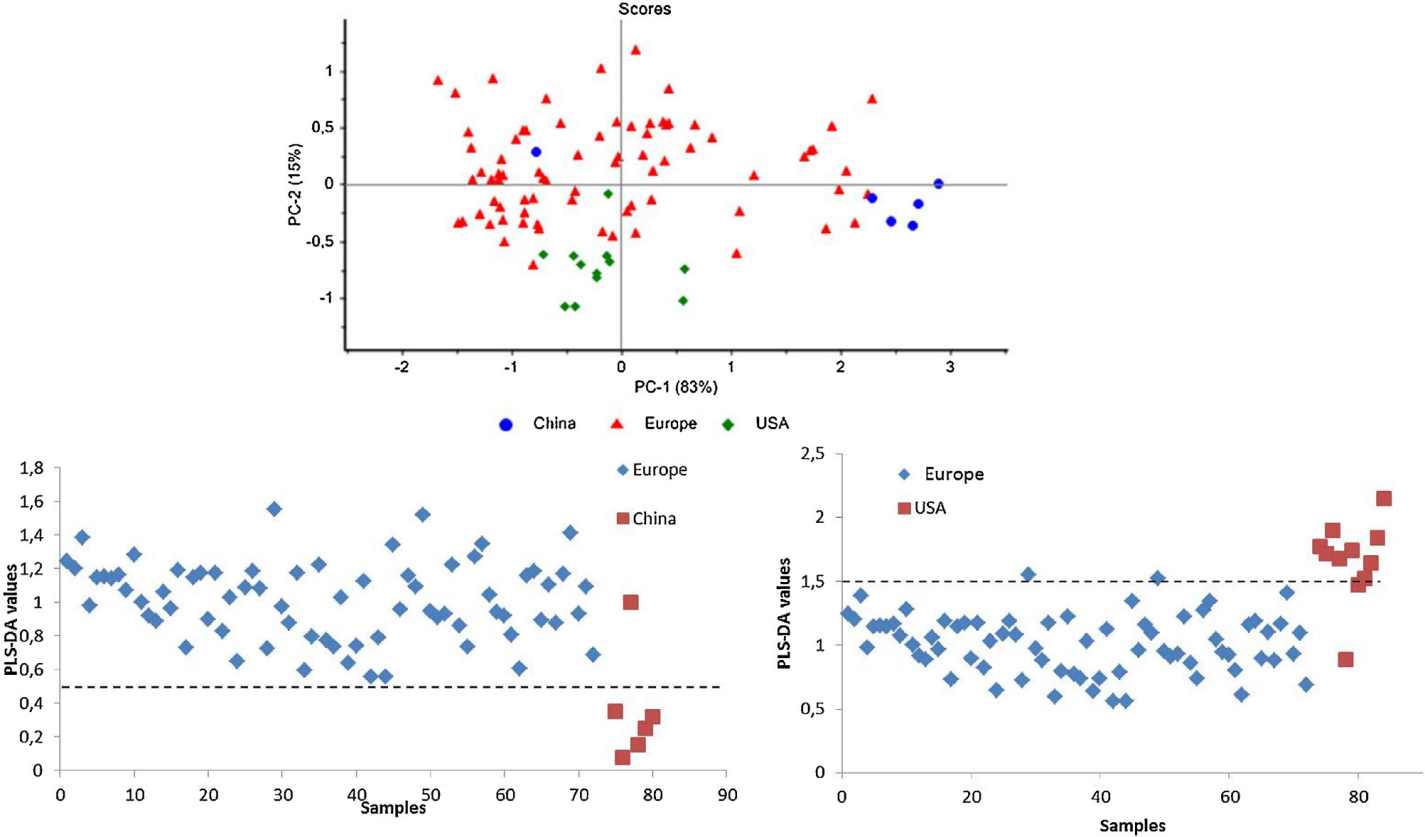
Score plot of PC1 versus PC2 of NIR spectra of Marlboro (PMI) tobacco (up), with cigarettes from Europe (red triangles), United States of America (green diamonds) and China (blue dots). Sample grouping of PLS-DA with Marlboro tobaccos from different origins, (a) China vs Europe (bottom-left); (b) Europe vs USA (bottom-right). (For interpretation of the references to colour in this figure legend, the reader is referred to the web version of this article.)

Among all the cigarette samples used in this study Marlboro Red were the most abundant (Table 2). Thus, Marlboro Red samples originating from Europe, China and USA were selected in order to build models for discriminating the geographical origin within a trademark. Spectra obtained from tobacco pellets from China (6 packs), USA (11 packs) and Europe (89 packs) were pooled in a data matrix and analysed by PCA (Fig. 4, up). PC1 and PC2 explained 83 % and a 15 % of the total variance, respectively, this PCA shows a trend among the samples according to their geographical origin: Europe, China and USA plotted in red, blue and green respectively. This trend is reinforced by the finding of one of the samples from China plotted in the middle of the European cloud. This sample was purchased in a duty free shop of an airport in China, what corroborates the fact of finding this sample among European samples.

Even if a trend is observed in the PCA, given the limited number of samples available for creating the model, the samples are projected mixed together, thus, as it was done before with the trademarks or companies, PLS-DA was carried out to confirm the discriminatory power of the model. The response variables were set to a value of 0 for Chinese samples; to a value of 1 for European samples and to a value of 2 for North American samples. In general terms, a clear separation between compared origins was achieved as observed in Fig. 4 (bottom). The models were obtained by employing the first 4 factors, subjected to random cross validation (15 segments). The trend observed in the PCA (Fig. 4, up) can be confirmed by means of the PLS-DA. In the discrimination analysis between China and Europe (Fig. 4, bottom-left), one Chinese sample is projected among European samples, which corresponds to the cigarette pack bought at the airport in China mentioned before. When analysing samples from USA and Europe by means of PLS-DA (Fig. 4, bottom-right), 4 samples are misallocated, which could already be predicted from the blurry area in the PCA. Despite th is fact, the PLS-DA confirms the trend observed in the PCA of Fig. 4, however, the amount of samples representing the geographical origins remains a limitation factor since the robustness of the models increases with the number of samples.

### 3.3 Discrimination of European and Chinese tobacco from several producers

**Fig. 5:**
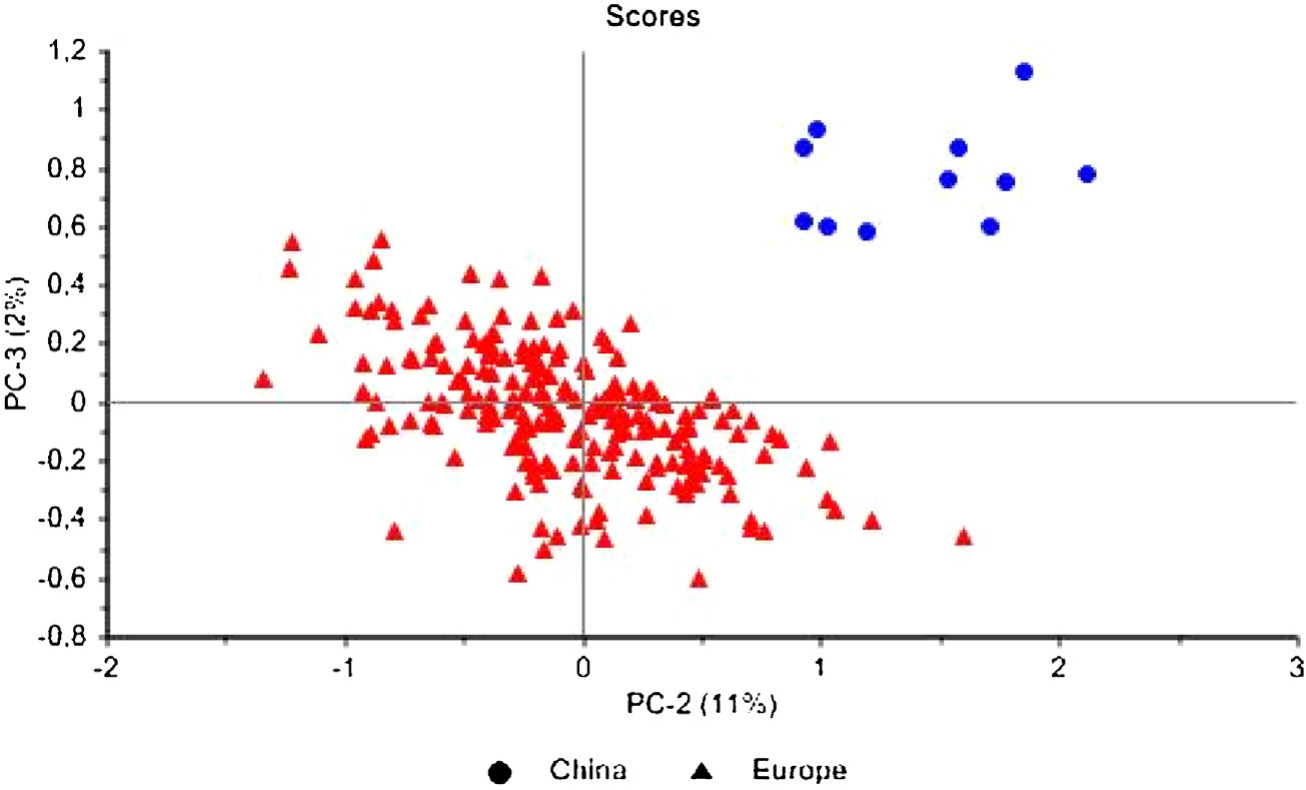
Score plot of PC2 versus PC3 of NIR spectra of European tobacco (red triangles) and Chinese tobacco (blue dots). (For interpretation of the references to colour in this figure legend, the reader is referred to the web version of this article.)

The same approach followed for achieving the geographical origin discrimination within a single tobacco trademark, was applied for the European samples of Marlboro Red, Camel and Lucky Strike and the 11 less known tobacco trademarks from China. The obtained PCA explained only 13 % by PC 2 and PC3, which means that subtle differences in the tobacco can allow the distinction of tobaccos, as it can be observed in Fig. 5. The less known Chinese samples (blue dots) are clearly separated from the cigarettes of European origin.

### 3.4 Detection of counterfeit cigarettes

**Fig. 6:**
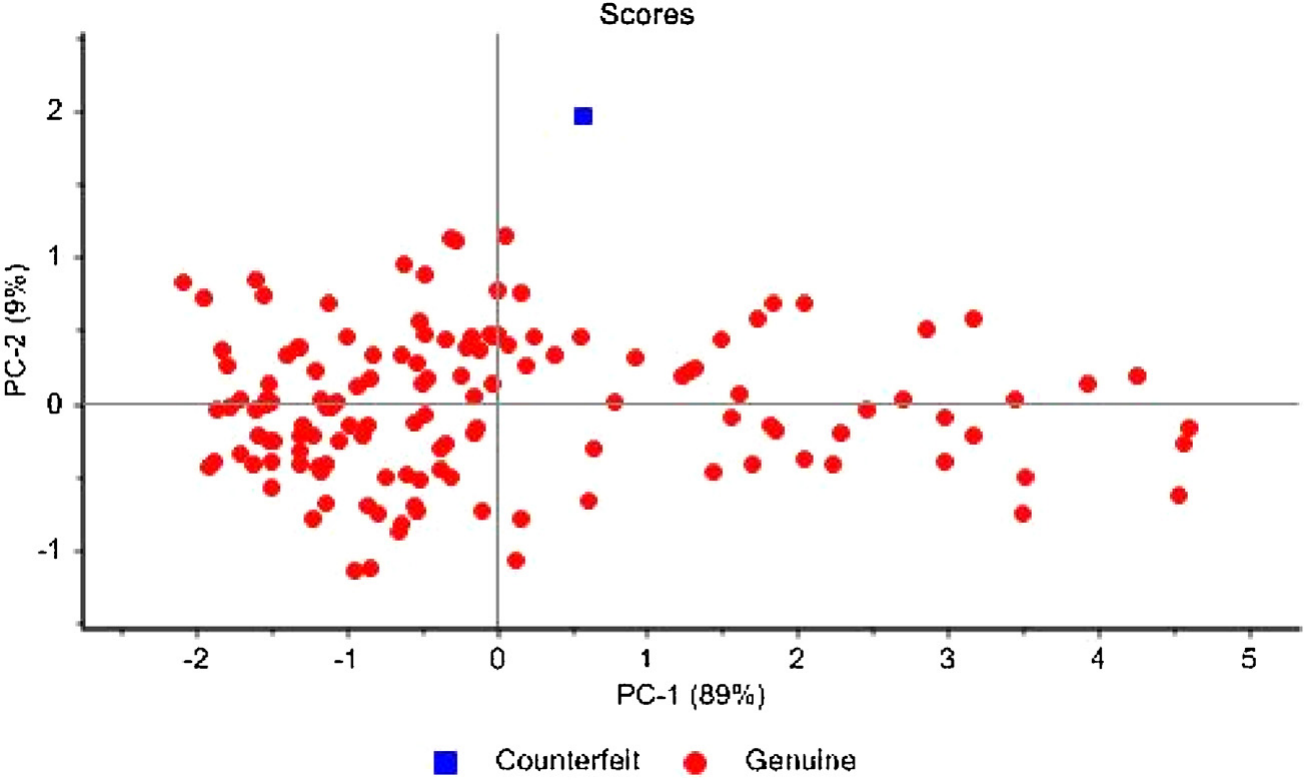
Score plot of PC1 versus PC2 of NIR spectra of European tobacco (red dots) and the counterfeited sample (blue square). (For interpretation of the references to colour in this figure legend, the reader is referred to the web version of this article.)

The same principle applied before for creating the models to differentiate among trademarks or producers was used to distinguish counterfeit from genuine tobacco. A PCA was carried out with all genuine EU and non-EU tobacco samples from PMI and a sample from the same trademark which was known to be counterfeited (Fig. 6). In this case the first two PCs of the PCA explained 97 % of the variance and the counterfeited sample was considered as an outlier at the 95 % confidence level. The described approach proved to be efficient to detect counterfeited samples from other well-known trademarks (results not shown for confidentiality reasons). The investigated suspect samples were projected together with genuine samples of the corresponding cigarette trademark present in the previously described repository and the suspicious samples were located outside the confidence ellipsis around the scores of the corresponding genuine samples.

## 4 Conclusions

This study presented an approach for the characterisation and discrimination of tobacco trademarks and/or producers by near infrared spectroscopy. The use of chemometrics is a key step in order to handle the data treatment and enhance the potential of the spectroscopic technique for classifying cigarette tobacco according to trademarks, geographical origin and authenticity.

The PCA and PLS-DA models generated by NIR spectroscopy with the tobacco pellets allow the distinction of cigarette trademarks and the identification of the producer. Moreover, the geographical origin of the samples can be differentiated between United States of America, China and Europe with the developed models. This information can be very useful for tracing trafficking routes and finding illicit products entering the countries. Moreover, the detection of counterfeit cigarettes was possible and the presented approach can be an important tool to support law enforcement authorities to detect illicit tobacco products.

This approach could be extended to other tobacco products and be used as non-destructive method for the discrimination of tobacco.

## CRediT authorship contribution statement

**Jone Omar**: Formal analysis, Methodology, Writing-original draft preparation, **Boleslaw Slowikowski**: Investigation, **Ana Boix**: Supervision, Writing-reviewing and editing.
